# Utility of a minimal skin incision technique for abdominal hysterectomy at a regional core hospital: a retrospective study

**DOI:** 10.1186/s13256-021-02715-7

**Published:** 2021-03-23

**Authors:** Wataru Isono, Masanori Maruyama

**Affiliations:** Department of Obstetrics and Gynaecology, Maruyama Memorial General Hospital, 2-10-5, Motomachi, Iwatsuki-ku, Saitama-shi, Saitama, 339-8521 Japan

**Keywords:** Minimal skin incision abdominal hysterectomy, Difficulty of operation, Blood loss, Operation time, Characteristics of leiomyoma

## Abstract

**Background:**

We present a minimal skin wound abdominal hysterectomy for patients with leiomyomas and describe the characteristics of this technique. The skin wound was made as small as possible, with a maximum length of 6 cm.

**Methods:**

In addition to introducing minimal skin wound abdominal hysterectomy, we retrospectively analyzed the medical records of 82 patients treated with minimal skin wound abdominal hysterectomy exclusively by two experts at Maruyama Memorial General Hospital between January 2013 and December 2016. Relationships between the leiomyoma characteristics and the difficulty of this operation, as estimated by operation time and blood loss, were statistically investigated.

**Results:**

First, we introduce a case in which we performed minimal skin wound abdominal hysterectomy on a 46-year-old Japanese patient with multiple leiomyomas (maximum 8 cm in diameter). Then, we assessed the impacts of the leiomyoma characteristics on the difficulty of this operation. On multivariate analysis, the number of leiomyomas significantly affected operation difficulty. Other characteristics of the target leiomyoma showed no effect. Additionally, higher body mass index also made the operation more difficult.

**Conclusions:**

Although multiple leiomyomas can make this procedure difficult, minimal skin wound abdominal hysterectomy is safe and effective for use in many cases.

## Background

Hysterectomy for patients with uterine leiomyomas is one of the most common gynecological surgeries [1], and all gynecologists in local private clinics and major regional or university hospitals often encounter these patients. Recently, less invasive technologies, including laparoscopic hysterectomy and other cutting-edge methods, have come to be the mainstream treatments [2]; however, the hard and soft capacities required are not necessarily available in all hospitals. In Japan, most hospitals with an obstetrics and gynecology department, especially those in rural areas, have severely limited human resources because most gynecologists must manage deliveries. Shorter operation time and reduced blood loss are crucial for hysterectomy. In contrast, large hospitals have many patients waiting for surgical treatments because of the large numbers of patients visiting these hospitals, which often exceed the hospitals’ capabilities. For patients with symptoms, especially massive vaginal bleeding, it is important to eliminate the long period between diagnosis and treatment. Accurate and varied information should be provided to help patients select their treatment methods. Therefore, we introduce our minimal skin incision laparotomy technique for hysterectomy, which offers improvements to the classical abdominal hysterectomy. In this procedure, we attempted to make the abdominal wound as small as possible, with a maximum length of 6 cm. In this retrospective analysis, we tried to identify the optimal application of our operation method in terms of decreased operative time and reduced blood loss.

## Methods

### Data collection

This study protocol was reviewed and approved by the Human Ethical Committee of Maruyama Memorial General Hospital (reference no. 2017-01). Subjects who underwent minimal skin incision abdominal hysterectomy (MAH) at Maruyama Memorial General Hospital between January 2013 and December 2017 were included, and the medical records of 82 patients with leiomyomas were reviewed retrospectively. In our hospital, two well-trained physicians performed abdominal hysterectomy to treat uterine leiomyoma while attempting to make as small a skin incision as possible. Therefore, the size of surgical skin scar was the only selection criterion of this study. All subjects completed MAH for uterine leiomyoma with skin incision less than 6 cm in length, and we excluded the cases in which we extended the skin incision greater than 6 cm. In these patients, the most frequent symptom was hypermenorrhea (55/82). We excluded patients who had other diseases, especially adenomyosis and endometriosis, as these would complicate the procedure because of the high possibility of severe adhesion. The size of surgical skin scar, operation time, and blood loss were recorded. As potential predictors of the level of difficulty for MAH, the number of leiomyomas and the size and location of the dominant leiomyoma were determined using magnetic resonance imaging (MRI) and ultrasound before surgery. The location of the dominant leiomyoma was classified within two categories, as follows: (1) anterior, posterior, or fundal leiomyomas; (2) intramural, submucous, or subserous leiomyomas. Total weight of the resected specimen from each patient was measured after surgery. Patient characteristics were also collected, including body mass index (BMI, kg/m^2^), age, administration of gonadotropin-releasing hormone analog (GnRHa), presence of hypermenorrhea, and presence of adhesion diagnosed during surgery.

### Statistical analyses

The statistical analyses were performed using JMP version 12 for Windows (SAS Institute, Inc., Tokyo, Japan). The categorical variables of the measured characteristics of the leiomyomas and other factors were compared to determine the correlations between these characteristics and the difficulty of MAH, estimated by operation time or blood loss. To eliminate confounding factors, we divided the patients into two groups according to the existence or nonexistence of each factor and used multivariate logistic regression analysis. For all patients treated with MAH, we assessed the influence of the following five factors, which included leiomyoma characteristics and BMI: (1) “higher BMI,” defined as BMI ≥ 25 (kg/m^2^); (2) “large leiomyoma,” defined as a dominant leiomyoma ≥ 8 cm; (3) “multiple leiomyomas,” defined as two or more leiomyomas; (4) “heavy uterus,” defined as a total weight of the specimen ≥ 500 g; (5) “adhesion,” diagnosed during operation. The criteria for “large leiomyoma”, “heavy uterus,” and “multiple leiomyomas” were determined based on past studies [3–6]. Because more than half of these patients (43/82) had multiple leiomyomas, total uterus weight was also compared. To compare the difficulty of operation, we defined the cases in which operation time exceeded 120 minutes or blood loss exceeded 400 ml as a difficult operation (DO), with these numbers determined by the average of the 82 cases. Operation time could not be compared with other studies because of differences in surgical skills, but the amount of blood loss was compared with past studies [7–9]. Odds ratios (ORs) and 95% confidence intervals (CIs) were estimated to determine the strength of these correlations. *p* < 0.05 was considered statistically significant.

### Surgical technique: minimal skin incision abdominal hysterectomy

**Fig. 1.**
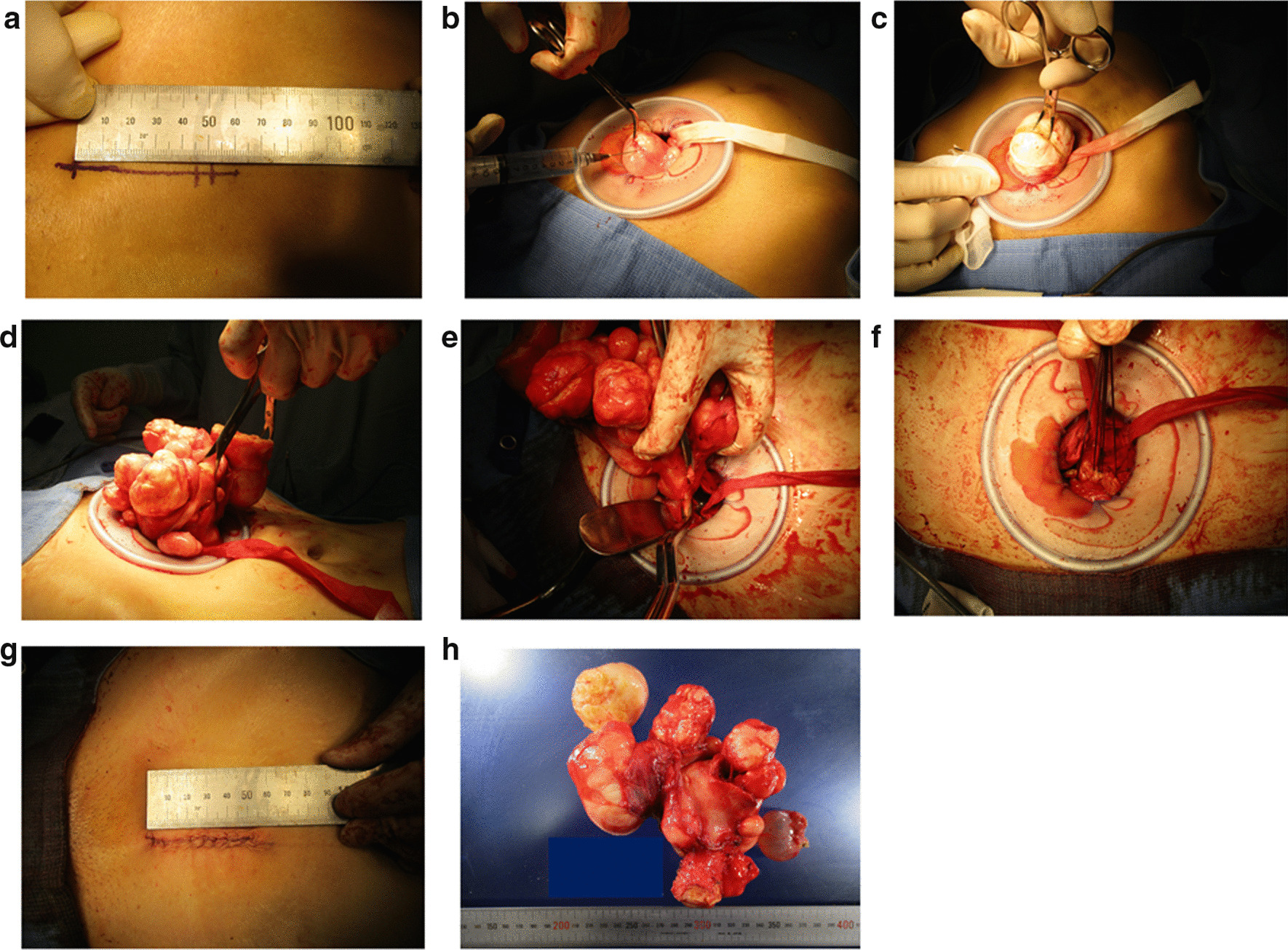
Surgical procedures of MAH. This patient was a 46-year-old Japanese woman with multiple uterine leiomyomas. The largest leiomyoma was over 10 cm in diameter on admission and was reduced to 8 cm in diameter after three rounds of GnRHa administration. She had a history of one gravidity and one parity. The operation time was 99 minutes, and the blood loss was 292 ml. The vertical incision was made in the middle of the abdomen 3 cm above the pubes. We resected the uterus and both fallopian tubes and preserved both ovaries. The total weight of the resected specimens was 467 g. **a** The size of the skin incision was 4.5 cm. **b** Appearance of the wound with medium Alexis wound protector/retractor and vasopressin solution injection into the surface of the uterus. **c** After several cuts to reduce its diameter, the leiomyoma was grasped with sharp clamps and pulled through the wound. **d** Appearance of the enucleation of the leiomyomas and the removed uterus. **e** The cut to the uterine artery. **f** The sutured vaginal stump. **g** Appearance of the sutured skin wound. After extending the wound with the aforementioned wound retractor, the skin wound reached a final length of 5.0 cm. **h** Appearance of the resected uterus and both tubes

All operations were performed exclusively by two physicians (M.M. and M.E.). All patients were operated on in the lithotomy position under general endotracheal anesthesia, and a Foley catheter was placed inside the bladder. Epidural anesthesia was also given. A skin incision less than 6 cm in length was made longitudinally (5.6 ± 0.5 cm, 4.0–6.0 cm, *n* = 82), and adipose tissue and abdominal fascia were cut using a monopolar electric scalpel (Fig. 1a). After the rectus abdominis muscle was spread, the peritoneal membrane was opened with a scalpel and surgical scissors. In this procedure, abdominal fascia and peritoneal membrane were cut longitudinally at a length of 8 cm. A medium Alexis wound protector/retractor (Applied Medical Resources Corporation, Rancho Santa Margarita, CA) was placed inside the wound to provide a wide operative view, making the uterus visible (Fig. 1b). Vasopressin solution (20 units in 100 ml normal saline) was injected into the surrounding tissue to decrease bleeding after the leiomyoma locations were detected. This represents approximately 2–3 ml injection, with each cut to the uterine trunk surface. The surface of the uterine trunk was cut with a monopolar electric scalpel, and a part of the leiomyoma was grasped with a sharp clamp (Fig. 1c). To pull the uterus through the wound in an upward direction without complete resection of each leiomyoma, a surgeon repeatedly cut and grasped different parts of the leiomyomas (Fig. 1d). By repeating this process, even a uterus that was much larger than the wound could be removed (Fig. 1e). After pulling the uterus out of the abdominal cavity, we cut the uterine artery (Fig. 1e) to complete the hysterectomy procedure. The vaginal wall wound was sutured with layered sutures within this scar (Fig. 1f), and the peritoneum, fascia, and skin were then sutured (Fig. 1g).

## Results

### Characteristics of patients treated with MAH

Average age of patients treated with MAH was 44.7 ± 3.6 years, and approximately 80% of all patients (64/82) were treated with GnRHa before surgery. Presence or absence of GnRHa use affected neither operation time (99.4 ± 14.1 versus 97.9 ± 16.6 minutes, *p* = 0.71) nor blood loss (187.5 ± 198.4 versus 129.3 ± 91.2 ml, *p* = 0.23). Average BMI was 22.3 ± 2.9 kg/m^2^. Average diameter of the dominant leiomyoma was 8.5 ± 3.1 cm (2.8–18 cm), and approximately half of all patients (39/82) were diagnosed with single leiomyoma. Average total weight of resected uterus was 451.1 ± 286.1 g (110–1800 g). Overall average operation time in the 82 patients was 99.0 ± 14.6 (68–142) minutes, and average blood loss was 174.8 ± 181.5 (2–1158) ml. First, we divided the 82 patients into two groups according to the size of skin scar (5.0 or less versus 5.5 or more) and compared the size of dominant leiomyomas between these groups. The average size was significantly larger in the latter group than in the former (*n* = 56, 8.9 ± 2.9 versus *n* = 26, 7.5 ± 3.3 cm, *p* < 0.05).

### Influence of five factors on operation difficulty

Based on the average of the 82 cases, a difficult operation (DO) was defined as the ten cases in which the operation time exceeded 120 minutes or blood loss exceeded 400 ml. In this analysis, we used the probability of DO, which was calculated by dividing the number of DO by the total number of cases. When evaluating the influence of leiomyoma location on operation difficulty according to two location classifications, no significant difference was detected in any of the following cases: (1) anterior: 13.3%, *n* = 4/30 versus posterior: 11.1%, *n* = 4/36 versus fundus: 12.5%, *n* = 2/16; (2) intramural: 14.8%, *n* = 8/54 versus submucous: 15.3%, *n* = 2/13 versus subserous: 0.0%, *n* = 0/15. Among the locations, only subserous leiomyomas showed a trend towards a reduced probability of DO (*p* = 0.11, versus submucous and *p* = 0.12, versus intramural). Presence or absence of GnRHa use also did not affect the probability of DO (14.1%, *n* = 9/64 versus 5.6%, *n* = 1/18, *p* = 0.33).

Next, the effects of the five factors were compared using multivariate analysis (Table 1). A significant difference was detected for cases with “multiple leiomyoma” (OR = 4.2, *p* < 0.01) or “higher BMI” (OR = 5.5, *p* < 0.01). “Large leiomyoma” (OR = 9.5, *p* = 0.072) and “adhesion” (OR = 3.0, *p* = 0.056) also showed a trend towards higher DO probability; however, “heavy leiomyoma” showed no significant effect (*p* = 0.68).

**Table 1.** Risk factors for increased difficulty of MAH

Factor	Number	OR (95% CI)	*p*-Value
Large leiomyoma	44	9.5 (1.1–79.0)	0.072
Multiple leiomyoma	43	4.2 (0.8–21.3)	<0.01
Heavy leiomyoma	28	3.4 (0.9–13.3)	0.68
Adhesion	23	3.0 (0.8–11.6)	0.056
Higher BMI	16	5.5 (1.4–22.4)	<0.01

Five factors were defined as follows: (1) “large leiomyoma,” defined as a dominant leiomyoma ≥ 8 cm; (2) “multiple leiomyomas,” defined as two or more leiomyomas; (3) “heavy uterus,” defined as total weight of the resected specimens ≥ 400 g; (4) “higher BMI,” defined as BMI ≥ 25 kg/m^2^; (5) “adhesion,” diagnosed during operation. The relationship between a difficult operation (DO), which was defined as an operation time exceeding 120 minutes or more than 400 ml of blood loss, and these five factors was assessed by multivariate analysis. In this analysis, both “multiple leiomyoma” and “higher BMI” were associated with a significantly higher probability of DO. In addition, “larger leiomyoma” and “adhesion” trended towards increased DO probability. In contrast, “heavy leiomyoma” showed no impact

## Discussion

According to current trends, laparoscopic hysterectomy is the most commonly performed procedure because faster recovery and a shorter hospital stay are expected in laparoscopic surgeries, and surgical wounds after laparoscopic surgeries are smaller than those after abdominal surgeries [10]. However, in rural areas, laparoscopic surgeries often cannot be performed because of shortages in skilled manpower and insufficient blood transfusion capacity [11]. Our hospital, Maruyama Memorial General Hospital, is located in a rural area of Japan, and abdominal surgeries are primarily performed for treating leiomyomas; therefore, we presented our procedure in this study as an alternative treatment option. On the analysis, we also detected some limitations deriving from the characteristics of a rural general hospital. Since almost all surgeries were performed by using longitudinal incisions in our hospital, we could not evaluate the outcomes of surgeries when using longitudinal or transverse incisions. The available data on postoperative status were also very limited; For example, the duration of hospital stay was uniformly set at 12–14 days for all patients with abdominal surgeries, and the cosmetic results could not be followed up in an outpatient setting. This will be the subject of a future study.

Combined with the techniques of myomectomy, we aimed to reduce the size of the skin scar and the degree of blood loss as much as possible. Two physicians (M.M. and M.E.) tried to make the skin incision less than 6 cm in length with ingenuity and originality, and a relatively short operation time (99.0 ± 14.6 minutes) and a decrease in blood loss (174.8 ± 181.5 ml) were achieved compared with past studies on laparoscopic hysterectomy [12, 13]. Therefore, the introduction of this method became one of the most important purposes of this study (Fig. 1). However, we failed to identify an objective indicator associated with skin scar size (4.0–6.0 cm), including the leiomyoma characteristics and patient BMI. This could be due to the relatively small sample size of 82 cases. The key aspects of the operational technique include the injection of vasopressin solution before cutting the uterine trunk surface to decrease bleeding, and the cutting of a part of the leiomyoma, which is then grasped with a sharp clamp to pull the uterus out of a small skin wound. The core of this method relies on repeated cutting and grasping of the leiomyoma to pull the uterus out from the abdominal cavity without complete enucleation of the leiomyoma (Fig. 1d). With its low cost and without the need for special equipment, this technique can be performed in any facility. However, one weakness of this technique is that its success mainly depends on the preexisting characteristics of the target leiomyoma. Therefore, the appropriate criteria for selecting this operational technique were also evaluated by performing multivariate analysis of several representative factors. On this analysis, we found that target leiomyoma number and size tended to increase the difficulty of MAH (Table 1). In particular, cases with “large leiomyoma” and “multiple leiomyomas” showed a relatively high probability of a DO at 33.3% (7/21). In contrast, the total weight of resected uterus did not affect the difficulty of the procedure. From our experience performing MAH, these results may be due to the following two reasons: (1) the repeated process of cutting and pulling the leiomyoma will increase operation time and blood loss, and (2) the difference between the skin scar size and the maximal length of target leiomyoma determines the difficulty of MAH. Because of the core feature of MAH, which is that the maximal length of the uterus is small enough for removal through a small wound, the maximal leiomyoma diameter is more important than the total uterus size. Moreover, the leiomyoma location did not affect the difficulty of the operation, probably because a relatively broad operative field derived from vertical incision was ensured to some extent. Along with target leiomyoma characteristics, our analysis also indicated that extremely obese patients may not be suitable for this method. In addition, the presence of severe adhesion detected during the operation might force operators to extend the initial small wound, likely because a thick fatty layer and adhesion result in a narrow operation field.

## Conclusions

These results cannot be easily generalized because of the many differences in target patient selection, the surgeons’ skill in performing hysterectomy, and other factors. However, with its short operation time and low blood loss, MAH was found to be safe and effective in many cases.

## Data Availability

The authors agree to make all data of this study freely available.

## Abbreviations

MAH: Minimal skin incision abdominal hysterectomy
BMI: Body mass index
GnRHa: Gonadotropin-releasing hormone analog
DO: Difficult operation
OR: Odds ratio
CI: Confidence interval
